# “The 24th Conference” of the Groupement des Pharmacochimistes de l’Arc Atlantique (GP2A)

**DOI:** 10.3390/ph10010017

**Published:** 2017-01-28

**Authors:** Jean-Jacques Hélesbeux, Olivier Duval

**Affiliations:** SONAS, SFR4207 QUASAV, University of Angers, 42 Rue Georges Morel, 49070 Beaucouzé, France; jean-jacques.helesbeux@univ-angers.fr

**Keywords:** medicinal chemistry, natural products, cancer, infectious diseases, tools

## Abstract

The GP2A European Conference is a two-day meeting focused on medicinal chemistry and the use of tools to explore all fields of drug discovery and drug design such as molecular modelling, bioorganic chemistry, MS studies, in vitro in vivo assays, and structure activity relationships. Abstracts of keynote lectures, plenary lectures, junior lectures, flash presentations, and posters presented during the meeting are collated in this report.

## 1. Aim and Scope of the Meeting

In 2016, the 24th Conference of the Groupement des pharmacochimistes de l’Arc Atlantique (GP2A) was held in Angers, at the Saint Serge Campus of the University of Angers (France), (25–26 August).

The main topics of the GP2A conference were natural products, anti-infectious agents, anticancer drugs, tumor-targeted nanosystems, and novel tools.

The scientific program included plenary lectures given by distinguished experts in the field, as well as oral and poster presentations by young scientists. Besides being a forum for discussions and scientific exchange, the GP2A organizers wish to provide a platform for young scientists to present their research.

## 2. Conference

### 2.1. Resistance by Allostery: A New Perspective for Eg5-Targeted Anticancer Drugs

McKaySimonStrathclyde Institute of Pharmacy and Biomedical Sciences, 31 Taylor Street, Glasgow G1, UK; simon.mackay@strath.ac.uk

Development of drug resistance during cancer chemotherapy is one of the major causes of chemotherapeutic failure for the majority of clinical agents. The aim of this study was to investigate the underlying molecular mechanism of resistance developed by the mitotic kinesin Eg5 against the potent second-generation ispinesib analogue SB743921, a phase I/II clinical candidate. Biochemical and biophysical data demonstrate that point mutations in the inhibitor-binding pocket decrease the efficacy of SB743921 by several 1000-fold. Surprisingly, the structures of wild type and mutant Eg5 in complex with SB743921 display no apparent structural changes in the binding configuration of the drug candidate. Furthermore, ITC and modelling approaches reveal that resistance to SB743921 is not through conventional steric effects at the binding site, but through reduced flexibility and changes in energy fluctuation pathways through the protein that influence its function. This is a phenomenon we have called “resistance by allostery”.

### 2.2. Natural Products and Chemical Ecology: Lichens as an Illustration

BoustieJoëlÉquipe PNSCM, Produits Naturels, Synthèses et Chimie Médicinale, Institut des Sciences Chimiques de Rennes, UMR CNRS 6226, U.F.R. des Sciences Pharmaceutiques et Biologiques Université de Rennes 1, Campus Villejean, 2, Avenue du Professeur Léon Bernard, 35043 Rennes CEDEX, France; joel.boustie@univ-rennes1.fr

Natural products remain a valuable source of medicines and biodiversity offers a great opportunity to discover new structures and new lead compounds. As an example of new natural sources providing additional opportunities through unique secondary metabolites, lichens represent one of these challenging sources. However, they are still poorly investigated, particularly as specific skills in a multidisciplinary approach are required. These mutualistic organisms belong to the fungi kingdom and they synthesize very unique and active compounds due to their symbiotic living form. The challenge is to study their metabolites from field observation and selective material collection to pharmacological properties through phytochemistry and chemical optimization. As poor data can be retrieved from their traditional medicine uses, interactions between the various partners within the lichen thallus can be informative for the properties of their secondary metabolites. Environmental interactions are also a key point to be studied, taking into account the combination of the multiple partners (phytobiont(s), mycobiont and associated microorganisms). Holistic approach of lichens which constitute a self-containing ecosystem are facilitated by new analytical developments as DART-MS and LDI-MS imaging from which the lichen thallus mapping of the secondary metabolites can be visualized.

### 2.3. Laser Desorption Ionization for the Detection of Small Molecules: A Powerful Complement to Classic LC-MS Approaches

SchinkovitzAndreas^1^*JaberAli^1^,^2^BoisardSéverine^1^KochAnnika^3^DreisewerdKlaus^3^SeraphinDenis^1^RichommePascal^1^1EA 921 SONAS/SFR 4207 QUASAV, University of Angers, 42 Rue Georges MOREL, 49070 Beaucouzé, France2Laboratoire de Recherche et Développement des Médicaments et des Produits Naturels RDMNP, Université Libanaise, Faculté de Pharmacie, Hadath, P.O. Box 6573, Beyrouth, Lebanon3Institut für Hygiene, Biomedizinische Massenspektrometrie, Westfälische Wilhelms-Universität Münster, Robert-Koch-Straße 51, 48149 Münster, Germany*Correspondence: andreas.schinkovitz@univ-angers.fr

In recent years metabolomic profiling and dereplication have become important tools in the field of natural products’ (NPs) research and quality control. These experiments are mainly based on methods such as high-performance liquid chromatography coupled with mass spectrometry (HPLC-MS) as well as ^1^H and ^13^C nuclear magnetic resonance spectroscopy (NMR) (El-Elimat, T., et al. *J. Nat. Prod.* 2013, *76*, 1709–1716; Williams, R.B., et al. *Org. Biomol. Chem.* 2015, *39*, 9957–9962)*.* On the other hand matrix assisted matrix laser desorption ionization (MALDI) and matrix free laser desorption ionization (LDI) are hardly discussed in this context. This lecture will highlight some recent advances in both methods such as the development of selective MALDI matrices for the detection of alkaloids in complex mixtures but also the matrix free LDI of NPs. Many NPs exhibit close structural similarities to MALDI matrices and can be directly ionized by LDI (Schinkovitz, A., et al. *J. Mass Spectrom.* 2015, *50*, 270–274*;* Le-Pogam, P., et al. *Anal. Chem.* 2015, *87*, 10421–10428). The effect is observed for a large variety of different compounds belonging to various chemical families, such as flavonoids, coumarins, tocopherols, and alkaloids, some of which could even be detected from crude extracts. Eventually the concept of selective signal enhancement by multi-wavelength laser irradiation targeting absorption maxima of selected analytes will be discussed.


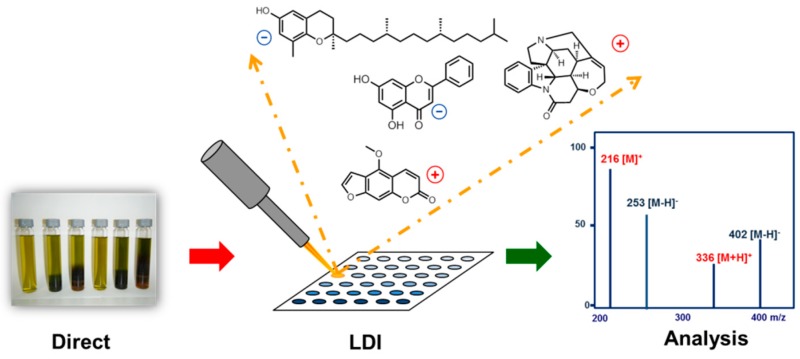


### 2.4. Identification of Novel Hit and Lead Compounds Inspired by Nature

StuppnerHermannInstitute of Pharmacy/Pharmacognosy and Center for Molecular Biosciences Innsbruck, University of Innsbruck, Innrain 80-82, 6020 Innsbruck, Austria; hermann.stuppner@uibk.ac.at

The treatment of inflammatory disorders in Western medicine relies heavily on the use of non-steroidal anti-inflammatory drugs (NSAIDs) and corticosteroids (Wang, Q., et al. *J. Ethnopharmacol.* 2013, *146*, 9–39). However, currently available treatment options are often unsatisfactory. Natural products (NPs) have always been an important source of new drug leads. Almost half of the drugs currently in clinical use are of natural product origin and even today, in the post genomic era, plants, fungi, marine organisms, and microorganisms are still an important source for the development of new drugs (Newman, D.J., et al. *J. Nat. Prod.* 2010, *75*, 311–335). 

In the course of a national research network project involving scientists of six Austrian universities we aimed to identify and characterize anti-inflammatory NPs capable to combat inflammatory processes specifically in the cardiovascular system. The combined use of computational techniques with traditional knowledge, high-tech chemical analysis, and synthesis, and a broad range of in vitro, cell-based, and in vivo pharmacological models led to the identification of a series of promising anti-inflammatory drug lead candidates. Mechanistic studies contributed to a better understanding of their mechanism of action and delivered new knowledge on the molecular level of inflammatory processes. Highlights of this interdisciplinary project which started in 2008 will be presented (Waltenberger, B., et al. *Monatsh. Chem.* 2016, *147*, 479–491).

**Acknowledgments:** This work was financially supported by grant n° S107 “Drugs from Nature Targeting Inflammation” from the Austrian Science Fund (http://www.uibk.ac.at/pharmazie/pharmakognosie/dnti/).

### 2.5. Riboflavin-Based Amphiphiles for Tumor-Targeted Nanosystems

BeztsinnaNataliia^1^TsvetkovaYoanna^2^SoleMarion^1^LammersTwan^2^KiesslingFabian^2^Berque-BestelIsabelle^1^*1Université de Bordeaux, UMR CNRS 5248 “Chimie et Biologie des Membranes et des Nanoobjets” “Molecular Modeling for (Bio) Molecule Engineering” MMBE, Allée Geoffroy Saint Hilaire, Bât B14 33600 Pessac, France2Experimental Molecular Imaging, RWTH Aachen University Clinic, 52074 Aachen, Germany*Correspondence: isabelle.berque-bestel@u-bordeaux.fr

Riboflavin (RF) or Vitamin B2 is essential for cell growth and development (Yao, Y., et al. *J. Nutr.* 2010, *140*, 1220–1226). It is internalized by the cells through a specific pathway, which involves a family of transporter (SLC52) and carrier proteins (RF carrier protein—RCP) (Johnson, T., et al. *Front. Biosci. Landmark Ed.* 2009, *14*, 3634–3640). The latter was found to be overexpressed in certain tumor cells as well as neovasculature. Thus addressing RF transporter systems represents a promising strategy for the generation of tumor-targeted drug carriers. 

The aim of this project is to develop nanosystems based on RF transporters to favor tumor targeting and cell uptake. These platforms consist in FDA approved liposomal formulations decorated by original tailor-made amphiphile RF derivatives and an encapsulated chromophore—rhodamine B for fluorescence microscopy and flow cytometry in vitro or indocyanine green (ICG) for non-invasive photoacoustic imaging in vivo (Beztsinna, N., et al. *Biomaterials* 2016, *80*, 121–133).

The cornerstone of this study is the preparation of tailor-made amphiphile derivatives of RF. These amphiphilic building blocks could be used as a versatile tool to develop targeting lipid based nanomedecines via RF transporter systems. 

These derivatives were inserted into liposomes leading to functionalized platforms. For the proof of concept, in cellulo investigations were performed on different cells: PC3, A431 cancer cell lines and HUVECs (Beztsinna, N., et al. *Bioconjugate Chem*. 2016, in proof).

Then, in vivo investigations in mice with A431 subcutaneous xenographs revealed the increase in targeted liposomes accumulation at early time points by photoacoustic imaging (VevoLAZR).


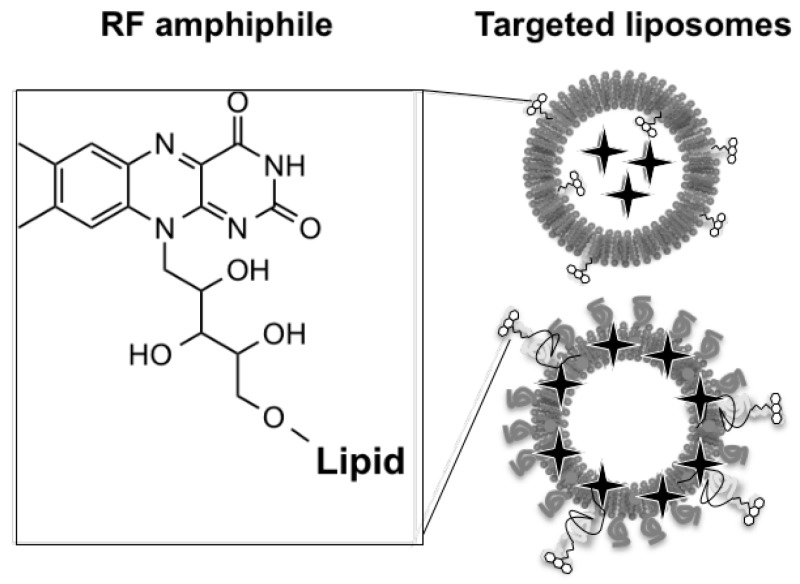


### 2.6. Anti-Adhesives: Future Therapy for Bacterial Infections?

MatthewsSusan E.School of Pharmacy, University of East Anglia, Norwich NR4 7TJ, UK; susan.matthews@uea.ac.uk

The challenge of increasing bacterial resistance to classical antibiotics is leading to the development of alternative strategies for treating bacterial infections. Anti-adhesive molecules that inhibit the interaction between bacterial and human cells offer an intriguing possibility for reducing bacterial load without a bactericidal effect.

This talk will focus on the development of anti-adhesives, based on macrocyclic glycoclusters, which inhibit two lectins from Pseudomonas aeruginosa (LecA and LecB) that are implicated in adhesion, biofilm formation and infectivity. The importance of ligand topology in achieving high binding strength and selectivity between different lectins will be described through comparison of libraries prepared from calix[4]arenes **1** (Cecioni, S., et al. *Chem. Eur. J.* 2009, *15*, 13232–13240; Sicard, D., et al. *Chem. Commun.* 2011, *47*, 9483–9845), resorcin[4]arenes **2** (Soomro, Z.H., et al. *Org. Bio. Chem.* 2011, *9*, 6587–6597) and pillar[5]arenes **3** (Buffet, K., et al. *Chem. Eur. J.* 2016, *22*, *9*, 2955–2963). Additionally, the potential modes of action and evaluation of the most effective inhibitor of LecA to-date in mouse model studies will also be discussed (Boukerb, A.M., et al. *J. Med. Chem.* 2014, *57*, 10275–10289).


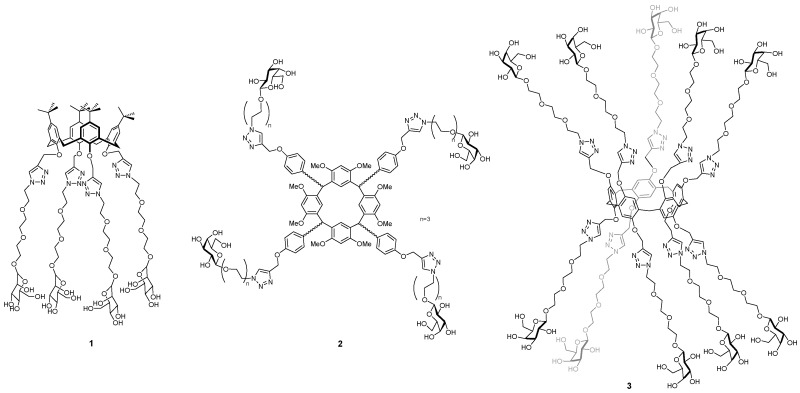


### 2.7. MR70, a Drug Candidate to Treat Malaria—From the Natural Hit to the Lead

CollotValérieCentre d’Etudes et de Recherche sur le Médicament de Normandie (UPRES EA 4258), Université de Caen Normandie, Boulevard Becquerel, CS 14032 Caen CEDEX 5, France; valerie.collot@unicaen.fr

*P. falciparum* malaria is the deadliest parasitic disease with 438,000 deaths in 2013. The emergence and the increasing proportion of *P. falciparum* parasites resistant to artemisinin derivatives, the most potent antimalarials, is a major concern in Southeast Asia. Fast acting drugs, with unaltered activity versus the current multi-drug resistant strains are urgently needed to replace artemisinins. Previously, traditional remedies such as *Cinchona* bark or *Artemisia* aerial parts led to the discovery of the most potent antimalarials, bearing out that Nature is still an incredible source of original compounds. Following this approach, we are developing new synthetic antimalarial agents based on the structure of an active natural product. We isolated a biflavonoid from *Campnosperma panamense* (IC_50_ = 480 nM in vitro on *P. falciparum* K1 multi-resistant strain), and developed novel simplified synthetic analogs (MR series) with improved pharmacological and pharmacokinetic profiles. One of these compounds, MR70, is strongly effective on *P. falciparum* early blood stage in less than 6 h. Moreover, MR70 and its analog MR87, exhibit a partial in vivo antimalarial activity, reducing parasitemia by 35% and 70% respectively on day 4 in a murine model (*P. berghei* ANKA, 100 mg/kg for 4 days). The investigations of structure-activity relationship are still ongoing to further improve these results. As MR70 acts specifically on early ring stage, which has been associated to artemisinin resistance, we have assessed the in vitro susceptibility of Cambodian artemisinin-resistant isolates to MR70 and found no cross-resistance between MR70 and artemisinins. These findings make flavone derivatives a promising new class of antimalarials. Further investigation is needed to optimize MR70 activity and assess its efficacy against strains resistant to partner drugs, usually combined with artemisinin derivatives, like piperaquine, mefloquine, lumefantrine, and amodiaquine.

### 2.8. Orphan Pathway Activation in Fungi—A Route to Chemical Novelty

BertrandSamuelUniversité de Nantes, Groupe Mer, Molécules, Santé-EA 2160, 44035 Nantes, France; Biogenouest, Plateau Thalassomics, 44035 Nantes, France; samuel.bertrand@univ-nantes.fr

Natural products (NPs) are important sources of novel bioactive compounds. Although many industries have ceased or significantly reduced their NP drug discovery programs, NPs continue to be of interest to pharmaceutical companies (Newman, D.J., et al. *J. Nat. Prod.* 2012, *75*, 311–335). Indeed, nature provides a massive reservoir of organisms that produce potentially beneficial compounds to be discovered and explored (Zhu, F., et al. *Proc. Natl. Acad. Sci. USA* 2011, *108*, 12943–12948). The inventiveness of nature regarding the production of innovative and unusual molecular skeletons is unmatched. Among the established sources of NPs, microorganisms (Fungi, Bacteria…) are presently one of the most attractive source of NPs in drug discovery (Demain, A.L. *J. Ind. Microbiol. Biotechnol.* 2014, *41*, 185–201; Lam, K.S. *Trends Microbiol.* 2007, *15*, 279–289), mainly because of their ubiquitous occurrence, their extensive biodiversity, and the large chemodiversity that can be found within a given species.

However, under classical laboratory growth conditions the full potential of fungal NPs chemodiversity is underestimated as most of secondary metabolites genes cluster remain silent (Pettit, R.K. *Microb. Biotechnol.* 2011, *4*, 471–478). To overcome such difficulties several approaches such as non-targeted metabolic engineering, epigenetic modification or elicitation, and the production of unnatural–natural scaffolds have recently been developed (Bertrand, S., et al. *Biotechnol. Adv.* 2014, *32*, 1180–1204). Such approaches lead to the induction of novel compounds by the activation of cryptic biosynthetic pathways dedicated to the production of secondary metabolites.

To monitor such induction, liquid chromatography coupled to high resolution mass spectrometry metabolomics (Wolfender, J.-L., et al. *J. Chromatogr. A* 2015, *1382*, 136–164) is now considered as a very efficient approach to study the induction of metabolite biosynthesis in fungi. This presentation will focus on exemplifying the use of metabolomics to track activation of orphan pathways through OSMAC (One Strain Many Compounds) approach (Bode, H.B., et al. *ChemBioChem* 2002, 3, 619–627), kinetics study (Roullier, C., et al. *Mar. Drugs* 2016, *14*, 103–117) and co-culture strategy (Bertrand, S., et al. *Biotechnol. Adv.* 2014, *32*, 1180–1204; Bertrand, S., et al. *Mol. BioSyst.* 2014, *10*, 2289–2298). Such strategies are nowadays largely used in NP drug discovery processes.

### 2.9. The Discovery and Development of Highly Selective Β1-Adrenoceptor Antagonists: A Safer Treatment for Patients with Concomitant Cardiovascular and Respiratory Disease?

KellamBarrieSchool of Pharmacy, Centre for Biomolecular Sciences, University of Nottingham, University Park, Nottingham NG7 2RD, UK; barrie.kellam@nottingham.ac.uk

β-blockers reduce mortality and improve symptoms in patients suffering from heart disease (CIBIS-II *Lancet* 1999, *353*, 9–13; MERIT-HF *Lancet* 1999, *353*, 2001–2007; Packer, M., et al. *Circulation* 2002, *106*, 2194–2199). However, current clinically available β-blockers display poor selectivity for the cardiac β1-adrenoceptor over the lung β2-subtype. Unwanted β2-blockade can result in life-threatening bronchospasm and reduce the efficacy of β2-agonist emergency rescue therapy (Lewis, R.V., et al. *Drug Saf.* 1993, *9*, 272–279). Hence people with concomitant heart disease and asthma are often denied these life-prolonging therapeutic agents through differing prescribing perceptions (Jones, T.E., et al. *Intern. Med. J.* 2013, *43*, 507–512). Here we describe a medicinal chemistry program which has resulted in the development of two highly β1-selective neutral antagonists with good pharmaceutical properties that can potentially overcome this limitation. They both display nanomolar β1-AR affinity, greater than 500-fold β1-AR vs. β2-AR selectivity and are orally bioavailable. 


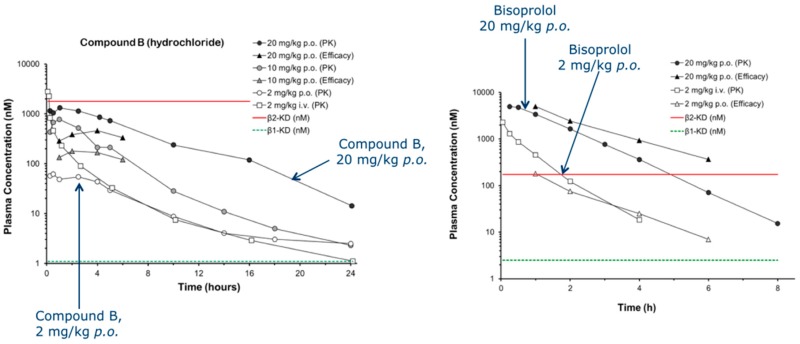


In addition, we have developed an hypothesis for their selectivity through in silico modelling. Both compounds elicit a β1-mediated reduction of heart rate in vivo, while displaying no effect on β2-mediated hindquarters blood flow. The compounds possess good ADME properties and have no adverse toxicological effects. They potentially offer a truly cardioselective β-blocker therapy for the significant number of people with concomitant heart and respiratory, or peripheral disease.

### 2.10. The Effect of Metal on the Sonodynamic Activity of Cationic Water-Soluble Porphyrin Complexes

GiuntiniFrancesca^1^*SerpeLoredana^2^FenoglioIvana^3^DurandoGianni^4^CanaparoRoberto^5^1School of Pharmacy and Biomolecular Sciences, Liverpool John Moores University, L3 3AF Liverpool, UK2Department of Drug Science and Technology, University of Torino, 10124 Torino, Italy3Department of Chemistry, University of Torino, 10124 Torino, Italy4INRIM (National Institute of Metrological Research), 10135 Torino, Italy5Department of Anatomy, Pharmacology and Forensic Medicine, University of Torino, 10124 Torino, Italy*Correspondence: F.Giuntini@ljmu.ac.uk

Sonodynamic therapy (SDT) is a therapeutic approach in which ultrasound irradiation is used to promote the generation of cytotoxic species via the excitation of particular chemical compounds (sonosensitizers). Similarly to photodynamic therapy (PDT), two individually non-toxic components initiate a process that culminates in the generation of reactive oxygen species (ROS), including radicals (hydroxy-, alkoxy-, and peroxy-radicals) and singlet oxygen, causing rapid intracellular damages and leading to cell death via apoptosis and/or necrosis and/or autophagy (Kuroki, M., et al. *Anticancer Res.* 2007, *27*, 3673–3677). The potential of this approach for the reduction of solid tumors has been shown, and its efficacy has been demonstrated at the preclinical level on some experimental tumor models (Serpe, L., et al. *Nanotech. Rev.* 2012, *1*, 173–182). Crucially, while light has a relatively limited reach within tissue, ultrasound easily propagates through it, allowing the targeting of deeply-seated lesions with minimal invasiveness invasive (Tachibana, K., et al. *Ultrasonics* 2008, *48*, 253–259).


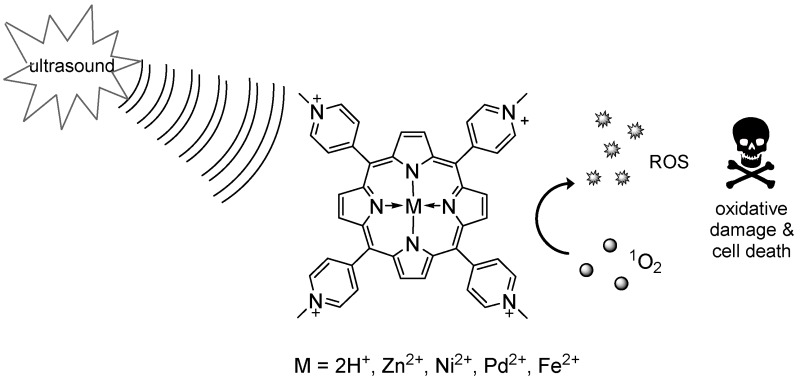


Despite these promising features, the scarce reproducibility of the treatment outcomes and the poor correlation between the in vitro and the in vivo results hampered progresses in the field: overcoming these disadvantages will require the elucidation of the molecular bases underlying sonosensitization, and the rationalization of its effects on cancer tissues. With this in mind, we undertook a study aimed at elucidating the effects of the presence and nature of the metal ion on the sonodynamic activity of a series of water-soluble porphyrin complexes. We will present the preliminary results of our investigation, concerning, in particular, the characterization of the ROS produced by the complexes under sonication and the preliminary results of sonocytotoxicity on HT-29 cells.

## 3. Poster Flash Presentations

### 3.1. Pd-Catalyzed and Copper-Assisted Sequential C2 andC7 Arylation of Potent Bioactive Thiazolo[5,4-f]quinazolin-9(8H)-ones

HarariMarineCoulyFlorenceFruitCorinneBessonThierry*Normandie University, UNIROUEN, INSA Rouen, CNRS, COBRA, 76000 Rouen, France*Correspondence: thierry.besson@univ-rouen.fr

Our research group focuses on the synthesis of C,N,S- or C,N,O-containing heterocyclic compounds as potential kinase inhibitors involved in Alzheimer’s disease (Schmitt, C., et al. *ACS Med. Chem. Lett.* 2014, *5*, 963–967; Dehbi, O., et al. *Eur. J. Med. Chem.* 2014, *80*, 352–363). Previous biological results led us to intensively study the thiazolo[5,4-f]quinazolin-9(8*H*)-one backbone for further SAR investigations (Hédou, D., et al. *Tetrahedron* 2013, 69, 3182–3191; Guillon, R., et al. *ACS Med. Chem. Lett.* 2013, *4*, 288–292; Hédou, D., et al. *Tetrahedron Lett.* 2015, *56*, 4088–4092). In this context, the late-stage modulation of this structure using C-H functionalization represents a very attractive approach (El Kazzouli, S., et al. *RSC Adv.* 2015, *5*, 15292–15327).

Based on previous work (Harari, M., et al. *Org. Lett.* 2016, *18*, 3282–3285; Laclef, S., et al. *Org. Lett.* 2015, *17*, 1700–1703; Godeau, J., et al. *Eur. J. Org. Chem.* 2015, *35*, 7705–7717), we developed an efficient method for the functionalization of the *N^8^*-benzylated thiazolo[5,4-*f*]quinazolin-9(8*H*)-one scaffold, based on selective C-H arylation. This strategy allows regioselective sequential arylation in the C2 and C7 positions via a judicious choice of the base and the coupling partners.


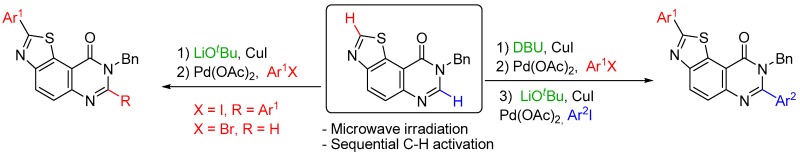


Diverse *N*^8^-benzylated-diaryl-thiazolo[5,4-*f*]quinazolin-9(8*H*)-ones and *N*^8^-benzylated-2-arylthiazolo[5,4-*f*]quinazolin-9(8*H*)-ones were thereby obtained. These procedures tolerate diverse aryl halides and would considerably facilitate the synthesis of libraries of potential kinase inhibitors.

### 3.2. Synthesis and Evaluation of Novel Benzofuran Conjugates as Potential Anticancer Agents 

McKeeMary L.*McCarthyFlorence O.Department of Chemistry and ABCRF, University College Cork, Western Road, T12 YN60 Cork, Ireland*Correspondence: m.mckee@umail.ucc.ie

The World Health Organization states that cancer is the leading cause of death worldwide, accounting for 8.2 million deaths in 2012 alone. (Stewart, B. W., et al. “World Cancer Report 2014”, 2014). Ellipticine (**1**) is a well-known anticancer agent with multiple modes of action. Since its discovery in 1959, both ellipticine and its derivatives have been extensively tested with the aim of generating a molecule with clinical applications. (Dalton, L., et al. *Aust. J. Chem.* 1967, *20*, 2715–2727). Research within our own group has shown discreet molecular modifications can lead to significant anti-cancer activity, including the synthesis of salts or derivatization of the A-ring (Deane, F.M., et al. *Org. Biomol. Chem.* 2013, *11*, 1334–1344; Russell, E.G., et al. *Investig. New Drugs* 2014, *32*, 1113–1122).

Our research looks to further probe the ellipticine template and alter the atoms in its very framework: what is the effect when a single nitrogen atom is replaced with an oxygen atom? The benzofuran moiety is present in numerous pharmaceuticals and has been shown to have important biological activity (Nevagi, R.J., et al. *Eur. J. Med. Chem.* 2015, *97*, 561–581). Our research employs the benzofuran moiety in an ellipticine type framework (**2**) to explore the biological potency of these molecules.


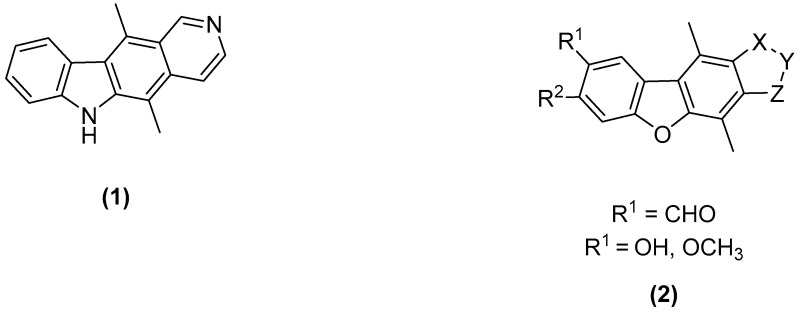


The core aims of this work are the synthesis of tetracyclic benzofuran derivatives with subsequent evaluation. The synthetic work presented here focuses on the functionalization of the D-ring of the molecule, generating novel D-ring analogues of ellipticine. Preliminary biological activity will also be reported.

### 3.3. New Series of Mcl-1/Bcl-xL Dual Inhibitors for the Treatment of Chemoresistant Ovarian Cancers

DenisCamille*LegayRémiBureauRonanVoisin-ChiretAnne-SophieNormandie Université, CERMN (Centre d’Etudes et de Recherche sur le Médicament de Normandie), UPRES EA 4258-FR CNRS 3038 INC3M, Bd Becquerel, F-14032 Caen, France*Correspondence: camille.denis@unicaen.fr

Protein-protein interactions play a fundamental role in many biological pathways as the apoptosis. Any disturbance of the apoptosis is connected with the phenomena of cancerization and resistance in the conventional chemotherapeutic treatments.

The control of the apoptosis involves the proteins of the Bcl-2 family (pro-apoptotic and anti-apoptotic members), interacting between them via a crucial helical BH3 domain.

Thus, the anti-apoptotic members (such as Bcl-xL or Mcl-1) inhibit the action of the pro-apoptotic ones by holding the BH3 domain of this last.

The strategy to release pro-apoptotic proteins is the use of small molecules, foldamers, able to mimic the BH3 domain of BH3-only proteins. Our laboratory has been interested for years in the synthesis of a large library of mixed (het)aromatic oligosystems as abiotic foldamers and made the proof of concept that such scaffolds were able to be BH3-mimetics with pro-apoptotic activity; particularly, our lab design, synthesize, and evaluate Pyridoclax as BH3-mimetics (Gloaguen, C., et al. *J. Med. Chem.* 2015, *58*, 1644–1668).


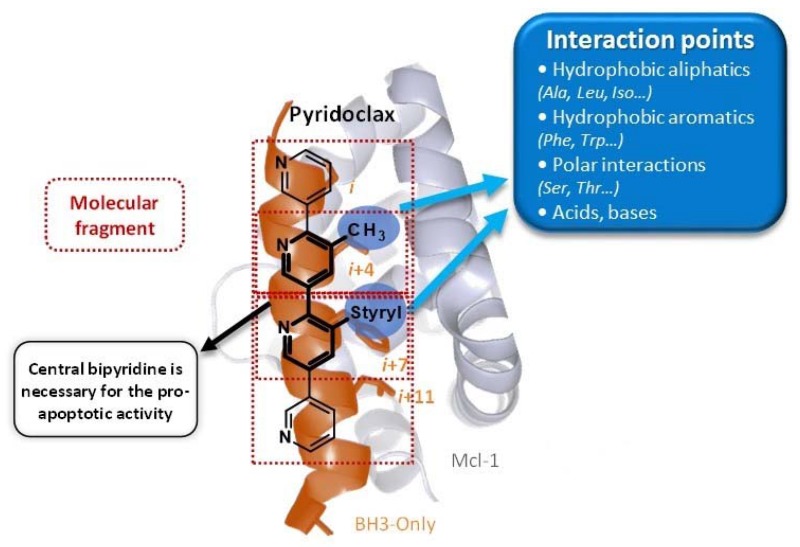


An effective treatment requires a simultaneous inhibition of several anti-apoptotic proteins of the Bcl-2 family. In this context, the discovery of dual inhibitors (Tanaka, Y., et al. *J. Med. Chem.* 2013, *56*, 9635–9645), acting on Bcl-xL and Mcl-1 could play an important role in the cancer treatment.

The strategy using Fragment-Based Methods and Structure-Based Design to design dual inhibitors from Pyridoclax will be presented.

### 3.4. Asymmetric Synthesis and Evaluation of a Range of Seco-Amino-Cbis as Highly Cytotoxic DNA Alkylating Agents

KennyMichael B. C.LloydMatthew D.ThompsonAndrew S.ThreadgillMichael D.*Medicinal Chemistry, Department of Pharmacy & Pharmacology, University of Bath, Claverton Down, Bath BA2 7AY, UK*Correspondence: prsmdt@bath.ac.uk

Duocarmycin SA **1** is a natural product of the CPI class with exquisitely potent cytotoxicity (IC_50_ = 10 pM in L1210 cells) (Ichimura, M., et al. *J. Antibiot.* 1990, *43*, 1037–1038). It binds to the minor groove of DNA, and alkylates using the strained spiro-cyclo-propane ring. Denny and others have shown that **2** is similarly super-potent; in this compound, the exocyclic carbonyl is replaced by an imine and the pyrrole ring is replaced by a second benzene ring, leading to the class being named amino-CBI (Atwell, G.J., et al. *J. Org. Chem.* 1998, *63*, 9414–9420; Twum, E.A., et al. *Bioorg. Med. Chem.* 2015, *23*, 3481–3489).

This work outlines a short, efficient, and asymmetric route to the correct enantiomer of the seco-amino-CBI. Starting from commercially available 1-hydroxynapthoic acid, seco-amino-CBI intermediate **3** can be synthesised in eight steps. At this point in the synthesis, a range of minor-groove binding subunits can be integrated after deprotection of the amine. This key intermediate is then transformed to the final seco-amino-CBI in a further three steps. As part of an exploration of the structure-activity relationship around the non-covalent minor-groove binding subunit, a short series of seco-amino-CBIs **4–6** were synthesized to be evaluated in vitro through MTS assays in LnCAP cells.


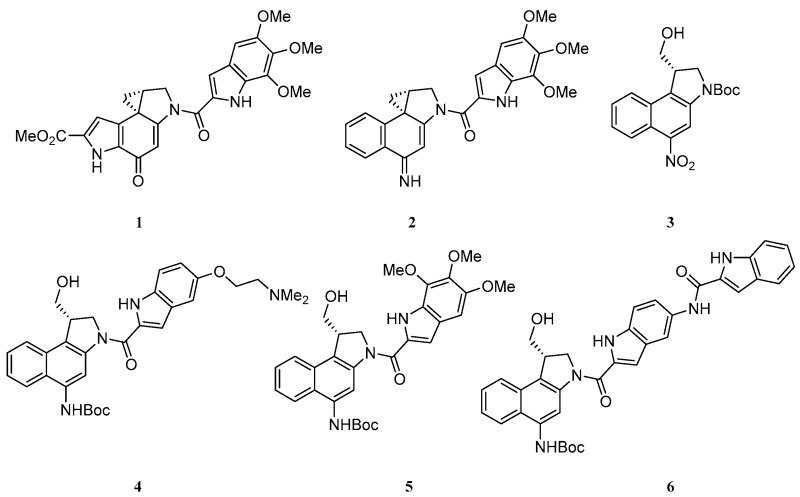


**Acknowledgments:** We thank Prostate Cancer UK for financial support.

### 3.5. Synthesis of Clx-BPA as Potential Endocrine Disruptor and Their Glucorono, Sulfo Analogues

DoumasManon^1^,^2^,^3^DupuisAntoine^1^,^3^,^4^RabouanSylvie^1^,^3^VenisseNicolas^3^,^5^Albouy-LlatyMarion^1^,^3^,^6^GrignonClaire^1^,^3^,^4^EugenePascale Pierre^1^,^3^MigeotVirginie^1^,^3^,^6^CaratoPascal^1^,^3^*1University Poitiers, UFR Médecine Pharmacie, 86073 Poitiers, France2IC2MP, CNRS 7285, 86073 Poitiers, France3CIC INSERM 1402, SEPEX, 86021 Poitiers, France4CHU Poitiers, Service de Pharmacie, 86000 Poitiers, France5CHU Poitiers, Service de Toxicologie et Pharmacocinétique, 86000 Poitiers, France6CHU Poitiers, Pole Biospharm Service de Santé Publique, 86000 Poitiers, France*Correspondence: pascal.carato@univ-poitiers.fr

For decades, studies of endocrine-disrupting chemicals (EDCs) have challenged traditional concepts in toxicology, in particular the dogma of “the dose makes the poison”, because EDCs can have effects at low doses that are not predicted by effects at higher doses. BPA is an endocrine disrupting chemical, specifically a selective estrogen receptor modulator. Numerous studies suggested that prenatal BPA exposure was correlated with preterm delivery (Miao, M., et al. *Reprod. Toxicol.* 2011, *32*, 64–68), low birth weight (Lee, B., et al. *Epidemiology* 2008, *19*, s365), and reduced head circumference (Snijder, C.A., et al. *Hum. Reprod.* 2012, *27*, 910–920) but also induced adult disease with reproductive disorders (infertility, endometriosis) (Rubin, B.S. *J. Steroid Biochem. Mol. Biol.* 2011, *127*, 27–34; Maffini, M.V., et al. *Mol. Cell. Endocrinol.* 2006, *254–255*, 179–186), neurobehavioral development and metabolic diseases (obesity, diabetes, heart disease, thyroid and liver function).

We focused our research on the determination of BPA and also their chloro BPA analogues (ClxBPA) which were formed during water chlorination. In vivo, these compounds could be oxidated, reduced or metabolized in glucuro or sulfo conjugated derivatives. For this proposal, a cohort of pregnant women was built (EDDS cohort) and human biological environments were collected like urine, human breast milk (colostrum) as well as also drinking water. 

Herein, for the first time, we describe the synthesis of ClxBPA and their conjugated analogues, as glucuro, sulfo derivatives.


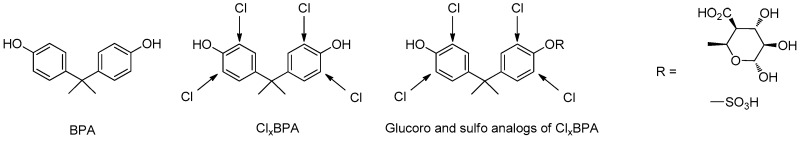


For a second time, these compounds were used as witness in an analytical study with LC-MS/MS to quantify them in human biological environments (urine, colostrum) and drinking water (Cariot, A., et al. *Talanta* 2012, *100*, 175–182; Migeot, V., et al. *Environ. Sci. Technol.* 2013, *47*, 13791–13797).

### 3.6. Display of Full Length Igg Antibodies on the Surface of Escherichia coli for the Construction of Antibody Libraries

DilkauteCarinaWeckenbrockWilhelmineJoseJoachimInstitute of Pharmaceutical and Medicinal Chemistry, PharmaCampus, Westfalian Wilhelms-University, Corrensstraße 48, 48149 Münster, Germany.*Correspondence: carina.dilkaute@uni-muenster.de

The number of antibodies used for therapeutic purpose is increasing continuously. For the discovery of new antibodies directed against a certain antigen, in most cases, phage display (Smith, G.P., *Science* 1985, *228*, 1315–1317) is used. Nevertheless, there are some disadvantages in using this method, such as the possible discrimination of the most potent binders during the biopanning process due to mild elution conditions. Furthermore, phage display is not compatible with flow cytometry and proteins displayed are limited in size (Levin, A.M., et al. *Mol. BioSyst.* 2006, *2*, 49–57). 

To circumvent these drawbacks antibodies were displayed on *E. coli* using the AIDA-I autotransporter (Jose, J., et al. *Microbiol. Mol. Biol. Rev.* 2007, *71*, 600–619). The presentation of antibodies and in particular antibody libraries enables the screening for new variants against pre-given epitopes using flow cytometry without losing the highly potent binders. Furthermore, the displayed proteins are not restricted in size. The screening can occur directly with full length antibodies, which are beneficial for therapeutic applications in most cases.

For proof of principle, the antibody T84.66 (Neumaier, M., et al. *Cancer Res.* 1990, *50*, 2128–2134) which is directed against the carcinoembryonic antigen (CEA) was investigated. The autotransporter fusion proteins of its light and its heavy chain were produced simultaneously in a single *E. coli* cell. To prove surface exposure a protease accessibility test was performed. Assembly of the heavy and the light chain was shown via co-immunoprecipitation of both chains and the functionality of the antibody was confirmed with a flow cytometry based antigen binding assay. The results confirmed the presence of heavy and light chains at the bacterial surface as well as their interaction to form a functional full length IgG antibody binding the antigen CEA.

The described technique of displaying antibodies on *E. coli* cells was used to display an antibody library at the bacterial surface. Therefore, the complementarity determining regions 3 (CDR3) of the heavy and the light chain were randomized separately using randomized oligonucleotides in a linear amplification reaction. After co expression of these mutated antibody chains, the resulting combinatorial library can be used for screening with new antigens from relevant targets e.g., from cancer.

### 3.7. Targeting Human Hyaluronidase Hyal-1 with Natural Compounds

LengersIsabelle^1^*OrlandoZoya^1^BrandtSimone^2^MelzigMatthias F.^3^BuschauerArmin^4^HenselAndreas^2^JoseJoachim^1^1Institute of Pharmaceutical and Medicinal Chemistry, PharmaCampus, Westfalian Wilhelms-University, Corrensstraße 48, 48149 Münster, Germany2Institute of Pharmaceutical Biology and Phytochemistry PharmaCampus, Westfälische Wilhelms-University, Corrensstraße 48, 48149 Münster, Germany3Institute of Pharmacy, Freie Universität Berlin, Königin Luise Str. 2+4, 14195 Berlin, Germany4Institute of Pharmacy, Department of Pharmaceutical/Medicinal Chemistry II, University of Regensburg, Universitätsstr. 31, 93040 Regensburg, Germany*Correspondence: isabelle.lengers@uni-muenster.de

The physiological and pathophysiological functions of the polysaccharide hyaluronic acid (HA) depend on its chain size. Space filling, anti-inflammatory, and antiangiogenic effects are triggered by high molecular weight HA (HMW HA) (>20 kDa). Its hydrolyzation by hyaluronidases leads to low molecular weight HA (LMW HA) (<20 kDa), resulting in inflammatory and angiogenic effects (Stern, R. *Semin Cancer Biol.* 2008, *18*, 275–280). Degradation of HA is mainly catalyzed by human hyaluronidase *Hyal 1*. It has been shown, that the expression level of *Hyal 1* is elevated in cancer cells, like prostate or bladder tumour cells (Lokeshwar, V.B., et al. *J. Urol.* 2000, *163*, 348–356; Lokeshwar, V.B., et al. *J. Biol. Chem.* 2001, *276*, 11922–11932). Therefore, *Hyal 1* appears to be an interesting target for drug discovery.

Surface display of active *Hyal 1* on *Escherichia coli* via Autodisplay enables the screening for potential inhibitors in a whole cell system. Based on this technique we determined the inhibitory effect of different plant-extracts and natural compounds on human *Hyal-1*. The IC_50_ values of *Malvae sylvestris flos*, *Equiseti herba*, *Ononidis radix* and *Althaea officinalis* were determined between 1.4 and 7.7 mg/mL. Furthermore, the IC_50_ values of four saponines were determined. The obtained IC_50_ value for glycyrrhizic acid, a known *Hyal-1* inhibitor, was 177 µM. The IC_50_ values for the identified novel inhibitors gypsophila saponin 2, SA1641, and SA1657 were 108 µM, 296 µM, and 371 µM, respectively (Orlando, Z., et al. *Molecules* 2015, *20*, 15449–15498). These extracts and compounds can be used as a starting point for the synthesis of new small molecule inhibitors targeting human *Hyal-1*. 

Extracts of *Althaea officinalis* are indicated for patients who suffer from dry cough to smooth mouth membranes. In an eukaryotic cell assay with a HaCaT keratinocyte cell line, the gene expression of *hyal-1* was determined after treatment with 125 µg/mL and 250 µg/mL of a root extract of *Althaea officinalis* for 24 h. HaCaT keratinocytes were chosen as a model cell line to represent skin and mucosal epithelial cells, considering the topical application of *Althaea officinalis* in pharmaceutical usage. It could be shown, that treatment with *Althaea officinalis* decreases expression of *hyal-1* gene in HaCaT cells by 30%. In conclusion, *Althaea officinalis* does not only inhibit the enzymatic activity of *Hyal-1* but also leads to a decrease in *hyal-1* expression.

### 3.8. Bioguided Isolation of Cytotoxic Compounds from Nocardia Ignorata, a Lichen-Associated Bacterium

NoëlAlba^1^*SoenGwendoline Van^1^RouaudIsabelle^1^FerronSolenn^1^HittiEric^2^TomasiSophie^1^1PNSCM, UMR CNRS ISCR 6226, UFR Sciences Pharmaceutiques et Biologiques, Université Bretagne Loire, 2 Av. du Professeur Léon Bernard, 35043 Rennes, France2INSERM UMR 1099,LTSI, 35042 Rennes, France*Correspondence: alba.noel@univ-rennes1.fr

Lichens are symbiotic organisms consisting of a fungus and an alga (Chlorophyta or Cyanobacteria), and are well-known as rich sources of novel compounds (Shukla, V., et al. *Phytochem. Rev.* 2010, *9*, 303–314. Shrestha, G., et al. *Phytochem. Rev.* 2013, *12*, 229–244). However, recent studies revealed the presence of bacterial microbiota associated with these organisms (González, I., et al. *Int. Soc. Microb. Ecol.* 2009, *3*, 1105–1115). These microorganisms may represent an underexplored reservoir of novel species of potential interest in the discovery of novel lead compounds. However, the biosynthesis of original bacterial compounds is influenced by cultivation conditions such as nutrients, incubation periods, pH, temperature, etc. Changing these parameters according to the OSMAC approach (Bode, H.B., et al. *Chem. Biol. Chem.* 2002, *3*, 619–627) (One Strain MAny Compounds) can result in different secondary metabolite profiles. Actinobacteria are known for their ability to produce compounds of clinical and pharmaceutical importance (Bérdy, J. *J Antibiot.* 2005, *58*, 1–26). Thus, we applied the OSMAC approach to *Nocardia ignorata*, which was isolated from *Collema (*Parrot, D., et al. *Sci. Rep.* 2015, *5*, 15839). The extracts obtained from various culture media (LB, TY, MB, ISP2) showed interesting cytotoxicity against two different cell lines. A statistical optimization of the production of active compound was established using Tagushi L9 orthogonal array design. The four selected parameters were temperature, pH, volume, and oxygenation of the medium with three different levels for each parameter. After several days of growth using a 5L bioreactor BioFlo^®^ 115, the culture medium TY was collected and extracted with XAD-7 resin yielding two extracts (supernatant and resin extracts). A bioguided isolation process showed that the cytotoxicity was focused in the supernatant (HaCaT (Human keratinocyte cells): IC_50_ = 20 ± 8 µg/mL; B16 (Murine melanoma cells): IC_50_ = 41 ± 8 µg/mL). This extract was then purified using flash chromatography, semi-preparative reversed-phase HPLC using a water/CH_3_CN gradient. This process led to the isolation of an active compound which is a novel bromide diketopiperazine identified by NMR and mass spectrometry data.


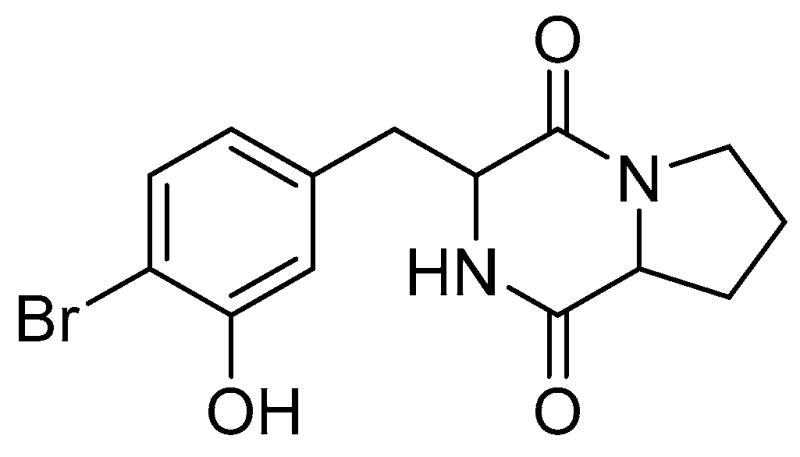


**Acknowledgments:** Delphine Parrot is acknowledged for bacterial isolation and preliminary assays. The “Ligue contre le cancer” is also acknowledged for financial support for the BioFlo^®^ 115 bioreactor.

## 4. Posters

### 4.1. Design, Synthesis, and Evaluation of the Cytotoxic Activity of New 3-{4-[(4-(Substituted)piperidin-1-yl)benzyl]}-2-Phenylindoles in Myeloid and Lymphoid Leukemia Cell Lines 

GuillonJean^1^*VincenziMarian^1^,^2^PinaudNoël^3^RongaLuisa^1^RossiFilomena^2^SavrimoutouSolène^1^MoreauStéphane^1^MarchivieMathieu^4^DesplatVanessa^5^1Université de Bordeaux, UFR des Sciences Pharmaceutiques, INSERM U1212/UMR CNRS 5320, Laboratoire ARNA, 146 Rue Léo Saignat, F-33076 Bordeaux CEDEX, France2Department of Pharmacy and CIRPeB, University of Naples “Federico II”, Via Mezzocannone, 16, 80134 Naples, Italy3Université de Bordeaux, ISM-CNRS UMR 5255, F-33405 Talence CEDEX, France4Université de Bordeaux, ICMCB CNRS-UPR 9048, 33608 Pessac CEDEX, France5Université de Bordeaux, UFR des Sciences Pharmaceutiques, Laboratoire BMGIC INSERM U1035, F-33076 Bordeaux CEDEX, France*Correspondence: jean.guillon@u-bordeaux.fr

Acute leukemia is one of the most aggressive hematopoietic malignancies and is characterized by the abnormal proliferation of the immature cells and a premature block in lymphoid or myeloid differentiation. Adult acute leukemia has a poor prognosis due to a large number of relapses, because of treatment-related resistance mechanisms (Lichtman, M.A. *Oncologist* 2008, *13*, 126–138).Therefore, there is an urgent need to find new therapeutics, which could led to the development of novel treatment strategies with less or minimal side effects. Heterocyclic compounds attracted a lot of attention because of their wide spread biological activities. Among them, the indole heterocyclic framework constitutes the basis of an important class of compounds possessing interesting biological activities.


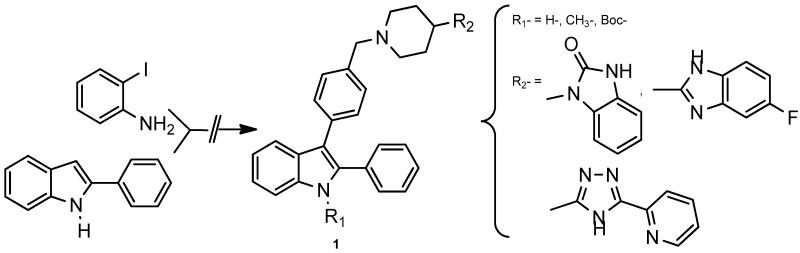


In the course of our work devoted to discover new compounds employed in cancer chemotherapy (Desplat, V., et al. *Eur. J. Med. Chem.* 2016, *113*, 214–227; Guillon, J., et al. *Struct. Chem. Crystallogr. Commun.* 2016, *2*, 18), we report herein on the synthesis and biological evaluation of new 3-{4-[(4-(substituted)piperidin-1-yl)benzyl]}-2-phenylindoles **1**. The cytotoxicity of these new derivatives was then evaluated against five different leukemia cell lines, including Jurkat and U266 (lymphoid cell lines), and K562, U937, HL60 (myeloid cell lines), as well as normal human peripheral blood mononuclear cells (PBMNCs). Biological results showed antiproliferative activities on the leukemia cell lines with IC_50_ in the μM range. In addition, some compounds are promising because of their high activity against leukemia (IC_50_ = 4–12 μM) and their low activity against normal hematopoietic cells (IC_50_ > 50 μM).

**Acknowledgments:** This work was supported by a grant from Ligue Nationale contre le Cancer (Comité Aquitaine-Charentes, Bordeaux, France).

### 4.2. Enantiopure Amino-Alcohol Fluorenes as New Antimalarial Drugs

SchneiderJérémy^1^*Dassonville-KlimptAlexandra^1^TaudonNicolas^2^SonnetPascal^1^1LG2A, CNRS UMR 7378, UFR de Pharmacie, Université de Picardie Jules Verne, 1 rue des Louvels, 80037 Amiens CEDEX, France2Unité de Toxicologie Analytique, Institut de Recherche Biomédicale des Armées, BP 73, 91223 Brétigny-sur-Orge, France*Correspondence: jeremy.schneider@etud.u-picardie.fr

Malaria is a neglected tropical disease that remains a leading cause of morbidity and mortality among the world’s poorest populations. More than 100 tropical and sub-tropical countries are endemic for this infectious disease. Pregnant women and children are the most sensitive to this infection and 438,000 people die of malaria each year (World Malaria Report, WHO, 2015, 2–81). Among the five species of *Plasmodium* responsible for human malaria, *P. falciparum* is the parasite which causes the most serious form of the disease. Unfortunately, this one is difficult to eradicate because of its capacity to develop varying degrees of resistance to many classes of antimalarial drugs. More recent efforts focused on the development of antimalarial vaccines and since 2006, the World Health Organization (WHO) has recommended artemisinin-based combination therapies (ACTs) (World Malaria Report, WHO, 2015, 2–81; 1—Guidelines for the treatment of malaria, 3rd edition, WHO, 2015, 214–219). In drugs resistance areas, several antimalarial drugs, such as amino-alcohol aryl (mefloquine (MQ), lumefantrine (LM)), are currently used in combination with artemisinin derivatives. However, the emergence of multi-drug-resistant parasites decreases the efficacy of ACTs making it crucial to find new active molecules against *Plasmodium*-resistant strains.

We previously developed a convergent and asymmetric synthesis to prepare 4-aminoalcohol quinoline enantiomers (AQ) as MQ analogs (Jonet, A., et al. *Tetrahedron* 2011, *67*, 128–143). They were active in the nanomolar range against 3D7 (chloroquine-sensible) and W2 (chloroquine-resistant) *Plasmodium falciparum* strains. Interestingly, (S)-enantiomers displayed an activity increased by 2 to 15-fold as compared to their (R)-counterparts (Mullié, C., et al. *Malar. J.* 2012, *11*, 65). These compounds, like amino-alcohol-aryl (MQ, LM), act on the intra-erythrocytic asexual stages but their mechanisms of actions remain to be explored (Mullié, C., et al. *Malar. J.* 2012, *11*, 65; Dassonville-Klimpt A., et al. Microbiology Book Series—2011, A Méndez-Vilas (Ed.), 2011, *1*, 23–35). 


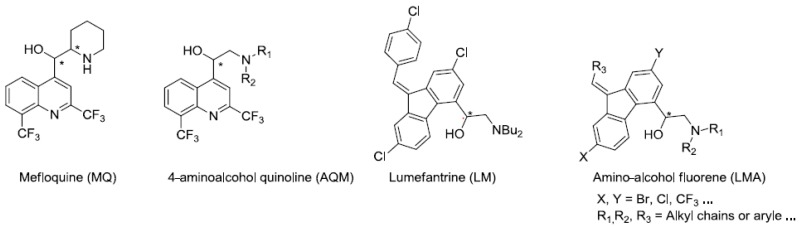


In continuation of our work on the study of the antimalarial activity and the mechanism of action of the amino-alcohol-aryl family, we are interested in studying the effect of the change of heterocycle (fluorene vs. quinoline) on the antimalarial activity. Herein, we describe the first steps of the enantioselective synthesis of new amino-alcohol fluorene derivatives (ALM) as LM analogs.

### 4.3. Synthesis of New Heterocyclic Compounds as Antifungal Agents and Biological Evaluation

DaoViet HungOurliac-GarnierIsabelleBazinMarc-AntoinePapePatrice LeMarchandPascal*Université de Nantes, Nantes Atlantique Universités, EA 1155-IICiMed, Institut de Recherche en Santé 2, 44200 Nantes, France*Correspondence: pascal.marchand@univ-nantes.fr

(−)-Cercosporamide was originally isolated in 1991 as an antifungal agent and phytotoxin from a fungal plant pathogen of cassava, *Cercosporidium henningsii* (Sugawara, F., et al. *J. Org. Chem.* 1991, *56*, 909–910).


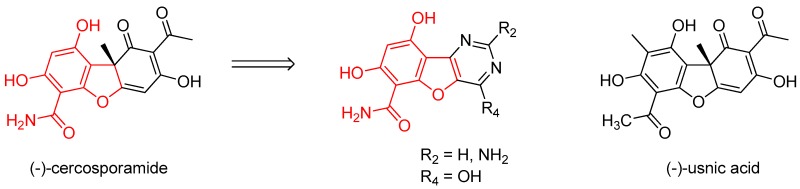


Its antifungal effect on *Candida albicans* strains results from its selective and potent inhibition of fungal PKC-like 1 kinase (Pkc1), which is central to cell wall integrity (IC_50_ = 44 nM for Candida Pkc1) (Sussman, A. et al. *Eukaryot. Cell* 2004, *3*, 932–943). Cercosporamide inhibition of human PKC isoforms PKCα, β and γ is less efficient (IC_50_ = 1.02, 0.35, and 5.8 µM, respectively). Moreover, CaPkc1 is supposed to be involved in the mechanism of antifungal drug resistance (LaFayette, S.L. et al. *PLoS Pathog.* 2010, *6*, 1–23).

The biological properties associated with cercosporamide, as a CaPkc1 inhibitor, are not found for its structural analogue usnic acid (Conover, M.A., et al. *Phytochemistry* 1992, *31*, 2999–3001), suggesting that the western part of cercosporamide is the pharmacophore of the molecule.

To design new molecules, this part was conserved and tricyclic analogues, bearing a pyrimidine moiety, were synthesized. To investigate the biological interest of these new molecules, their IC_50_ were determined, following the EUCAST procedure, on a collection of *Candida albicans* strains selected for their sensitivity or resistance to fluconazole (reference compound). In the present work, the synthetic routes and the first results of the biological evaluation will be discussed.

### 4.4. Synthesis of Purines in One or Two Steps from 5-Amino-imidazole-4-carbonitriles

BollierMélanie*KlupschFrédériqueMilletRégisLeleu-ChavainNataschaICPAL, Univeristy Lille, Inserm, U995-LIRIC-Lille Inflammation Research International Center, 3 rue du Pr Laguesse BP83, F-59006 Lille, France*Correspondence: melanie.bollier@etu.univ-lille2.fr

Imidazo[4,5-*d*]pyrimidines, better known as purines, are present in many natural health products. Purines play an important role in biological processes and due to their implication in many biochemical pathways, they have a wide therapeutic potential. Indeed, purines found applications as interferon inducers, antimycobacterial, leukotriene A4 hydrolase inhibitors, sulfotransferase inhibitors, phosphodiesterase inhibitors, adenosine receptor ligands, nucleoside transport inhibitors, kinase inhibitors, etc. Purines were synthesized for the first time by Hermann Emil Fischer from uric acid in 1898. Because of the large pharmacological interest in this heterocycle (Legraverend, M., et al. *Bioorg. Med. Chem.* 2006, *14*, 3987–4006), many synthetic routes to purines are described in the literature starting for instance from imidazole or pyrimidine precursors.

Herein, we report an original strategy to access rapidly a large diversity of purines. The advantage of our approach is to allow the construction of vast 8,9-disubstituted-purine libraries from 5-amino-1,2-disubstituted-1*H*-imiazole-4-carbonitriles.


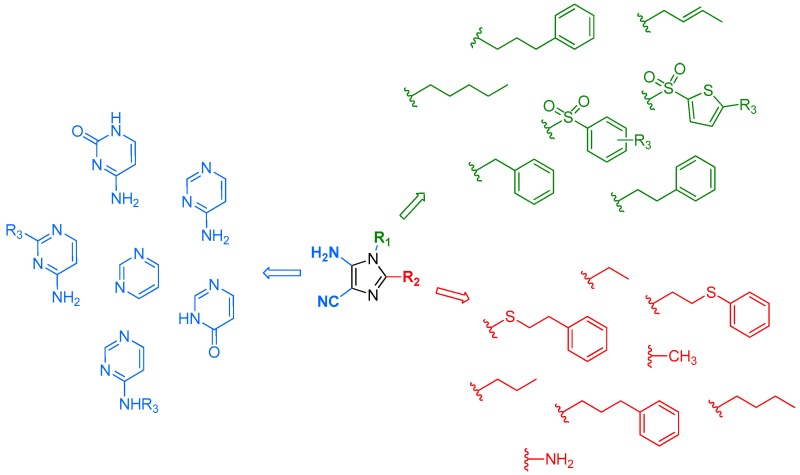


### 4.5. Pyridoclax, a BH3-Mimetic, Directly Inhibits Mcl-1 and Sensitizes Ovarian Cancer Cells to Bcl-xL-Targeting Strategies— From Design to Preclinical Evaluation

KiefferCharline^1^HedirSiham^2^SantosJana Sopkova-de Oliveira^1^JuinPhilippe^3^,^4^RaultSylvain^1^PoulainLaurent^2^Voisin-ChiretAnne Sophie^1^*1UNICAEN, CERMN-FR CNRS INC3M, 14032 Caen, France2UNICAEN, BioTICLA UMR 1199 INSERM, 14076 Caen, France; Comprehensive Cancer Center François Baclesse, 14076 Caen, France3Team 8, Nantes-Angers Centre for Cancer Research, UMR 892 INSERM-6299 CNRS, 44007 Nantes, France4Institut de Cancérologie de l’Ouest, Comprehensive Cancer Center R. Gauducheau, 44805 Nantes, France*Correspondence: anne-sophie.voisin@unicaen.fr

Apoptosis control defect such as the deregulation of Bcl-2 family members expression is frequently involved in chemoresistance. In ovarian carcinoma, we previously demonstrated that Bcl-xL and Mcl-1 cooperate to protect tumor cells against apoptosis and their concomitant inhibition leads to massive apoptosis even in absence of chemotherapy. Furthermore, Mcl-1 down-regulation or inactivation was required to sensitize cells to Bcl-xL-targeting strategies. Whereas Bcl-xL inhibition is accessible (using BH3-mimetics), Mcl-1 inhibition is problematic. In this context, we designed and synthesized oligopyridines potentially targeting Mcl-1 hydrophobic pocket, evaluated their capacity to inhibit Mcl-1 in live cells, and implemented a functional screening assay to evaluate their ability to sensitize ovarian carcinoma cells to Bcl-xL-targeting strategies. We established structure-activity relationships and we focused attention on MR29072, named Pyridoclax. Without cytotoxic activity as a single agent, Pyridoclax inhibits Mcl-1 in combination with Bcl-xL-targeting siRNA or with ABT-737 against ovarian, lung cancer cells and in mesothelioma (Gloaguen, C., et al. *J. Med. Chem.* 2015, *58*, 1644–1668).


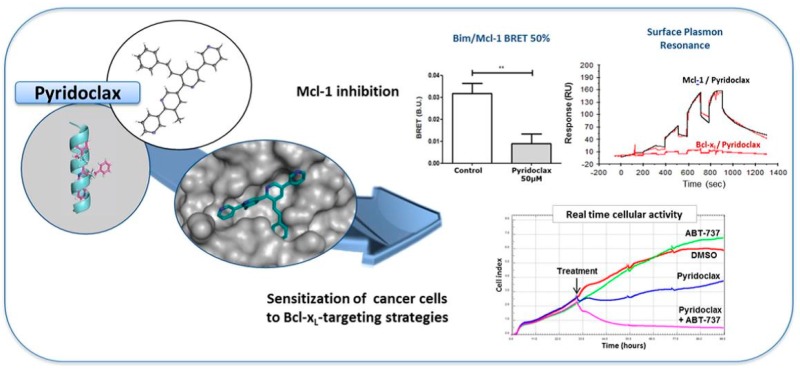


### 4.6. Bithiophenic Matrix for Selective Detection and Quantification of Toxic Alkaloids by MALDI-TOF-MS

JaberAli^1^,^2^*SchinkovitzAndreas^1^GuiletDavid^1^RichommePascal^1^SeraphinDenis^1^1Laboratoire SONAS-SFR QUASAV Substances d’Origine Naturelles et Analogues Structuraux, Campus de Végétal, 42 Rue Georges Morel, 49070 Beaucouzé, France2Laboratoire RDMPN-Recherche et Développement des Médicaments et des Produits Naturels, Université Libanaise, Faculté de Pharmacie, Hadath, P.O. Box 6573 Beyrouth, Liban*Correspondence: ali.jaber@etud.univ-angers.fr

Alkaloids are common secondary metabolites in plants and are often known for their wide range of pharmacological but also toxic effects. The compound 3-[5′-(methylthio)-2,2′-bithiophen-5-ylthio]propanenitrile (MT3P), has been used as a novel matrix molecule (Schinkovitz, A., et al. *Anal. Bioanal. Chem.* 2012, *403*, 1697–1705), which facilitates the selective ionization of alkaloids in matrix-assisted laser desorption/ionization time-of-flight mass spectrometry (MALDI-TOF MS). Therefore, MT3P might be used to develop a direct method of quantification of alkaloids in complex samples such as crude plant extracts. Analytical parameters were evaluated in order to validate the quantification of alkaloids in four plant extracts by MALDI-TOF MS, with MT3P as a matrix.


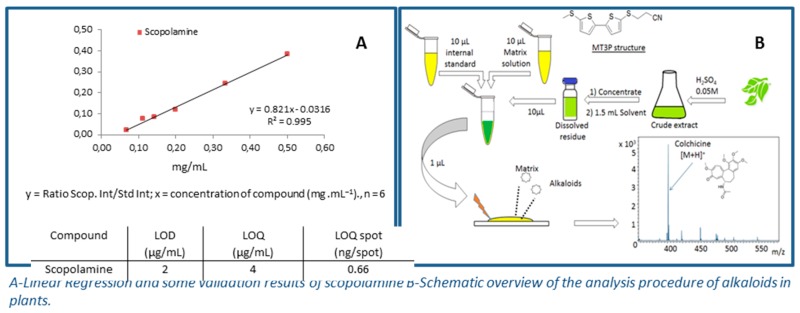


The proposed method was validated by assessing its repeatability, LODs and LOQs, linearity range, and accuracy. Depending on the tested alkaloid, the linearity was observed over the range between 50 and 500 μg, with a coefficient of correlation (R^2^) ≥0.993. The intra and inter-day variations ranged from 8.9% to 9.3% and 8.8% to 12.6%, respectively. The method was then successfully applied to direct profiling of alkaloids in several extracts of toxic plants, i.e., from the Solanaceae family. As no tedious chromatographic processes for separation were needed, this method can serve as an alternative to classical techniques used, such as LC-MS, for this type of study. In summary, M3TP was evaluated and applied to develop a rapid, sensitive, and selective method for the analysis of toxic alkaloids in complex samples, using MALDI-TOF MS.

### 4.7. Synthesis, Characterization, and Biochemical Evaluation of Novel Heterocyclic Stilbene Compounds with Tubulin Targeting Activity

AnaG.*O’BoyleN.CrowleyE.MeeganMary J.School of Pharmacy & Pharmaceutical Sciences, Trinity Biomedical Sciences Institute, Trinity College Dublin, Dublin 2, Ireland*Correspondence: anag@tcd.ie

Drug-like small molecule natural product such as colchicine and the combretastatins provide ideal structural templates for the design of structural scaffolds with improved efficacy as tubulin targeting agents. Combretastatin A-4 originally isolated from Combretum caffrum, exhibits strong antitubulin activity by binding to the colchicine binding site (Pettit, G., et al. *J. Med. Chem.* 1998, *41*, 1688–1695). In this work a library of amide conjugates of Combretastatin A-4 and related structures were synthesized and the biological activity investigated. We report our synthetic approaches to stilbene analogues of colchicines, combretastatins, and phenstatin together with their in vitro biological activity. We also report docking studies and the SAR of these compound classes as tubulin targeting agents, which have underlined that the presence of the 3,4,5-trimethoxy substituted A-ring and the 4-methoxy substituted B-ring separated by a double-bond to be essential for antiproliferative activity. The activity of CA4 is limited by isomerization of the active cis-stilbene configuration into the inactive *trans* analog. Introduction of a heterocyclic amide group on the stilbene scaffold may create a stabilized CA4-like compound locked in its cis-form.


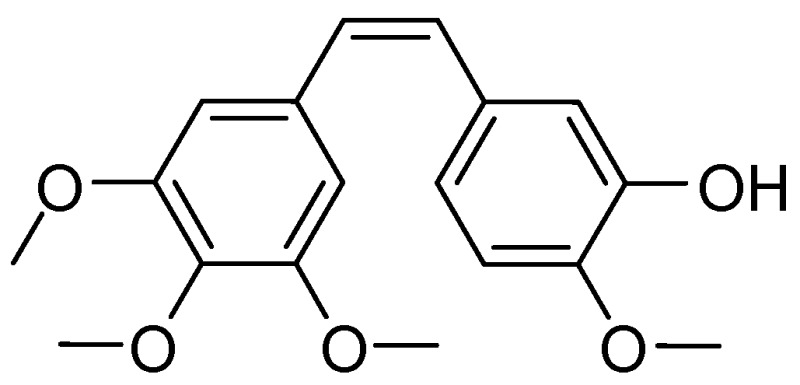


The first step in the synthesis of our library of stilbene derivatives is the Perkin reaction of an aromatic aldehyde and a substituted phenylacetic acid, to afford the acrylic acid combretastatin analogues. The reaction was optimized using a microwave reactor. The newly synthesized acrylic acids were then coupled to a variety of heterocycles using the Mukaiyama reagent as coupling reagent (using appropriate protecting/deprotecting steps) and purified via flash column chromatography to afford the heterocyclic amide conjugates. The novel compounds synthesized were characterized (^1^H-NMR, ^13^C-NMR, IR, HRMS). The purity of the final products was evaluated by HPLC and when possible the structure of the compounds was established by single crystal X-ray analysis. The biological activity was evaluated on the human MCF-7 cell line in an antiproliferative assay and the IC50 value for the most active compounds obtained.

### 4.8. Design, Synthesis, and Evaluation of Azetidin-2-Ones as Novel Bioactive Compounds

MalebariAzizah*MeeganMary J.School of Pharmacy & Pharmaceutical Sciences, Trinity Biomedical Sciences Institute, Trinity College Dublin, Dublin 2, Ireland*Correspondence: melibaa@tcd.ie

The African willow tree Combretum caffrum Kuntze (Combretaceae) is a very productive source of cancer cell growth (murine P388 lymphocytic leukemia) inhibitory stilbenes, bibenzyls, and phenanthrenes. The synthesis of a series of rigid analogues of combretastatin A-4 is described which contain the four membered β-lactam heterocyclic azetidinone in place of the usual ethylene bridge present in the natural combretastatin stilbene products (O’Boyle, N., et al. *J. Med. Chem.* 2010, *53*, 8569–8584). The structure-activity relationships of antiproliferative β-lactams, focusing on modifications at the 3- and 4-position of the β-lactam ring, are described. Synthesis of this series of compounds was achieved utilizing the Staudinger and Reformatsky reactions. To investigate the importance of the OCH3 substituent on C-4 of the aryl Ring B moiety for the biochemical activity and potency, a series of thioether S-CH_3_ and S-CH_2_-CH_3_ to replace the oxygen substituent at the C-4 position of the Ring B aryl moiety were synthesized, which were then evaluated for antiproliferative activity against three human cancer cell lines (MCF-7, HT-29, and HL60 cancer cell lines. Of a diverse range of heterocyclic derivatives, 3-phenyl, 3-vinyl, and 3-hydroxy analogues displayed the highest potency with nanomolar IC_50_ values, comparable to combretastatin A-4. A new class of combretastatin A-4 analogues that have thioether substituents at the C-4 position of the Ring B-aryl moiety of the β-lactam ring was successfully synthesized. All thioether S-CH_3_ compounds exhibited significant antiproliferative activity equally to -OCH_3_ β-lactams which can inhibit the first-pass metabolism pathway (formation of glucuronide metabolite) occurring by -OCH_3_ β-lactams.


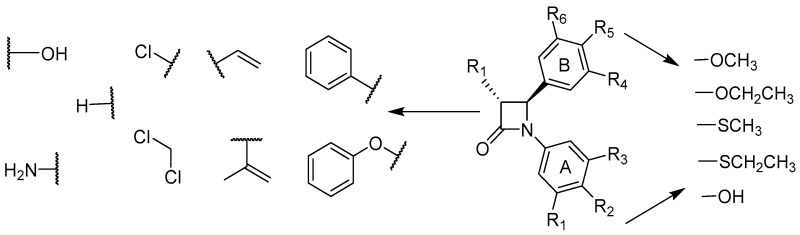


### 4.9. Discovery of Therapies for Lymphomas, and Leukaemias: Synthesis and Antiprolifertive Action of Novel Ethanoanthracenes

McKeownJames P.*ByrneAndrew P.MeeganMary J.School of Pharmacy & Pharmaceutical Sciences, Trinity Biomedical Sciences Institute, Trinity College Dublin, Dublin 2, Ireland*Correspondence: mckeowjp@tcd.ie

The antidepressant maprotiline and its (E)-9-(2-nitrovinyl)anthracene analogues were identified as potent novel antiproliferative compounds in Burkitt Lymphoma (BL) cell lines MUTU-1 and DG-75 (Cloonan, S.M., et al. *Int. J. Cancer* 2011, *128*, 1712–1723; McNamara, Y.M., et al. *Eur. J. Med. Chem.* 2014, *71*, 333–353). This knowledge was translated to CLL (Chronic Lymphocytic Leukaemia) a related B cell malignancy. CLL is the most common leukaemia in the western world, accounting for greater than one third of new diagnoses. 

Initial biochemical screening (Alamar Blue) in PGA1 and HG3 CLL cell lines was completed and a number of potent compounds were identified. The results were then compared to BL studies, with compounds being tested at 1 μM and 10 μM concentrations. Activity was determined as percentage of viable cells remaining. The subsequent finding of these studies is presented together with discussion of the rationalization of the compound SAR in PGA1 and HG3 CLL cell lines. Synthesis of (E)-9-(2-nitrovinyl)anthracenes was accomplished through the use of a piperidine catalyzed Henry-Knoevenagel condensation reaction, in addition to Grignard and Vilsmeier-Haack chemistry to yield the required 10-substituted 9-anthraldehyde intermediates.

Future work includes the expansion of the current SAR knowledge for the ethano-anthracene-type maprotiline analogues and bio-isosteric replacement of the nitro group on the 9-vinyl anthracene substituent with a view to optimizing biological activity.


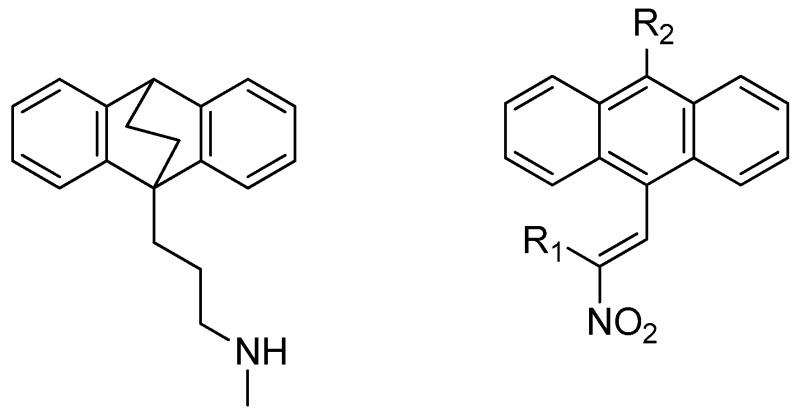


### 4.10. Synthesis and Evaluation of Novel Serotonin-4 Receptor Radiotracers for Single Photon Emission Computed Tomography

LalutJulien^1^TournierBenjamin B.^2^CaillyThomas^1^LecouteyCédric^1^*CorvaisierSophie^1^DavisAudrey^1^BallandonneCéline^1^MilletPhilippe^2^FabisFrédéric^1^DallemagnePatrick^1^RochaisChristophe^1^1Normandie University, UNICAEN, CERMN, 14000 Caen, France2Hôpitaux Universitaires de Genève, Département de Santé Mentale et de Psychiatrie, Service de Psychiatrie Générale, Unité des Biomarqueurs de Vulnérabilité, Chemin du Petit-Bel-Air, 2, CH-1225 Genève, Switzerland*Correspondence: cedric.lecoutey@unicaen.fr

One of the greatest challenges facing medicinal chemists in the 21st century is the discovery and development of diagnostic markers of Alzheimer’s disease (AD). The diagnosis of Alzheimer’s patients is usually made very late, using mainly tests like the Mini-Mental State Examination or Rey-Osterrieth’s figure. The establishment of an early diagnosis would allow an advanced support for a patient, potentially delaying the progression of symptoms.

Serotonin 5-HT_4_ receptors (5-HT_4_R) control brain physiological functions such as learning and memory, feeding, and mood behavior as well as gastro-intestinal transit. Several major devastating illnesses could benefit from 5-HT4 receptors-directed therapy such as AD (Bockaert, J., et al. *Curr. Opin. Pharmacol.* 2011, *11*, 87–93). 

Several 5-HT_4_ ligands synthesized in the laboratory were investigated as potential ligands for MTDL Alzheimer’s therapy (Lecoutey, C., et al. *PNAS* 2014, E3825–E3830; Lecoutey, C., et al. *Med. Chem. Comm.* 2012, *3*, 627–634) and for the brain imaging of 5-HT_4_R (Cailly, T., et al. *Eur. J. Med. Chem.* 2010, *45*, 5465–5467), using single-photon emission computed tomography (SPECT), and appear to be promising for medical applications. The 5-HT_4_R radiotracers must allow a quantification and a specific labeling of 5-HT_4_R rich regions as a reduction of 5-HT_4_ receptor density has been demonstrated in the brains of Alzheimer’s patients (Reynolds, G.P., et al. *Br. J. Pharmacol.* 1995, *114*, 993–998).

In the present project, we have chosen to present novel analogues of donecopride, a multi-target directed ligand based on the pharmacomodulation of a 5-HT_4_R partial agonist, as new potential radiotracers for SPECT imaging (Lecoutey, C., et al. *PNAS* 2014, E3825–R3830; Eglen, R.M., et al. *Br. J. Pharmacol.* 1995, *115*, 1387–1392). Compounds were radiolabeled with 125-Iodine by the neuroimaging unit of Geneva University Hospitals (HUG), and administered by intravenous injection to rats to obtain brain autoradiograms in vitro, in vivo, and ex vivo.


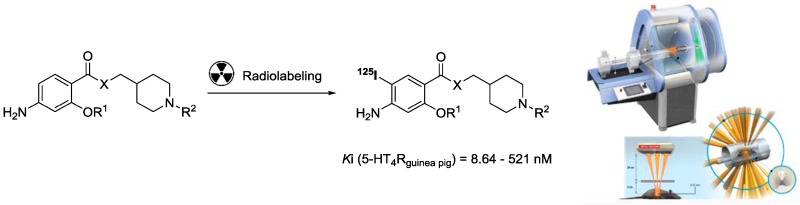


### 4.11. Synthesis and Biological Evaluation of a New Cathepsin C Inhibitor (XPZ-01)

CroixCécile^1^HamonYveline^2^ChoisySandrine Dallet^2^Viaud-MassuardMarie-Claude^1^*PedersenJohn^3^GauthierFrancis^2^KorkmazBrice^2^1UMR CNRS 7292 GICC Equipe 4 Innovation Moléculaire et thérapeutique, UFR des Sciences Pharmaceutiques, Université de Tours, 31 Avenue Monge, 37200 Tours, France2Centre d’Etude des Pathologies Respiratoires, INSERM U-1100, Faculté de Médecine de Tours, Université François Rabelais, Bât 47C, 10 Bld Tonnellé, 37032 Tours, France3CEO, CSO, M.Sc., UNIZYME Laboratories A/S, Dr. Neergaards Vej17, DK2970 Hörsholm, Denmark*Correspondence: marie-claude.viaud-massuard@univ-tours.fr

Neutrophil serine proteases (NSPs) contribute to the destruction of lung tissue in several inflammatory lung diseases, especially cystic fibrosis (CF) and chronic obstructive pulmonary disease (COPD). Attempts to inhibit these proteases in situ still remain unconvincing due to the protective environment made of mucus and DNA in bronchial secretions. These compounds compromise the efficient inhibition of proteases by exogenous therapeutic inhibitors. An alternative way is to control the activity of NSPs before they are delivered on inflammatory sites. This can be done by inhibiting cathepsin C (CatC), a cysteine protease that activates pro-NSPs in the bone marrow.

In this work, we describe the large scale synthesis (~5 g) and the biological evaluation of a CatC inhibitor ((S)-2-amino-N-((*1S,2S*)-1-cyano-2-(4’-(4-methylpiperazin-1-ylsulfonyl)biphenyl-4-yl)cyclopropyl)butanamide) first described as XPZ-01 by Unizyme Laboratories.


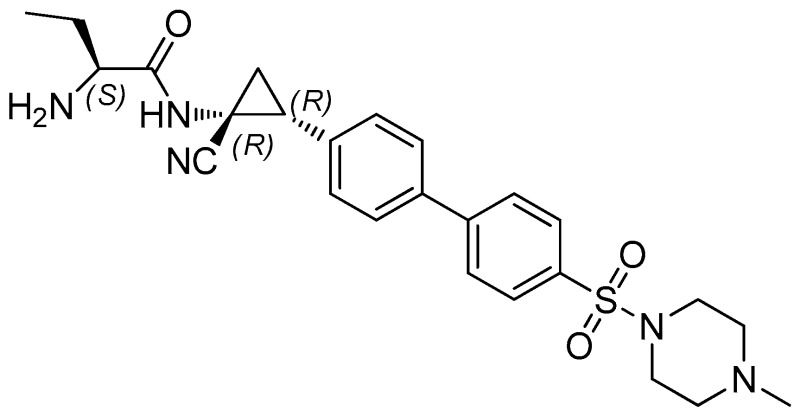


This inhibitor will be first tested on CatC extracted from the neutrophil of macaques since the animal model is the most relevant for preclinical studies on the fate of CatC during neutrophil-dominated inflammatory diseases.

### 4.12. δ-Garcinoic Acid, a Convenient Natural Precursor to the Attractive δ-Tocotrienol

VilleAlexia*ViaultGuillaumeHélesbeuxJean-JacquesSéraphinDenisSONAS EA921, SFR QUASAV 4207, Université d’Angers, 42 Rue Georges Morel, 49070 Beaucouzé, France*Correspondence: alexia.ville@univ-angers.fr

Vitamin E includes eight chemically distinct chromanols named α, β, γ, δ-tocopherols (T) and tocotrienols (T3). They mainly differ by the number and the position of aromatic methyl groups and by the nature of the side chain, phytyl for T and farnesyl for T3 (Ahsan, H., et al. *Nutr. Metab.* 2014, *11*, 52–73). For a long time, α-T was considered to be the most active form of vitamin E. However, recent researches suggested T3 to be better antioxidant agents (Theriault, A., et al. *Chem. Biochem.* 1999, *32*, 309–319). Moreover, T3 showed hypocholesteromic, anti-cancer, and neuroprotective properties that are not exhibited by T (Ahsan, H., et al. *Nutr. Metab.* 2014, *11*, 52–73; Theriault, A., et al. *Chem. Biochem.* 1999, *32*, 309–319). Compared with T, T3 have been less studied. 

δ-T3 was found in several plants such as *Mammea neurophylla*, *Hevea brasiliensi,s* and *Bixa orellana*. Nevertheless, either its purification from easily available plant materials is tedious because of the presence of all the vitamin E isoforms (*Bixa orellana*, *Hevea brasiliensis*) or the plant materials with a high δ-T3 content are difficult to obtain (Lavaud, A., Ph.D. dissertation, Université d’Angers, 2012). For instance, it was purified as the only vitamin E form with 0.5% yield from the barks of *Mammea neurophylla*, an endemic tree from New Caledonia (Dang, B.T., et al. *Fitoterapia* 2014, *96*, 65–75).

On the other hand, large amounts of garcinoic acid (1%), a T3 derivative, from the seeds of *Garcinia kola*, are isolated. This source is a renewable material mostly used in traditional medicine and known as bitter kola (Terashima, K., et al. *Heterocycles* 1997, *45*, 1559–1566). 

Therefore, we thought that the semisynthesis of δ-tocotrienol from garcinoic acid could be an attractive alternative pathway.


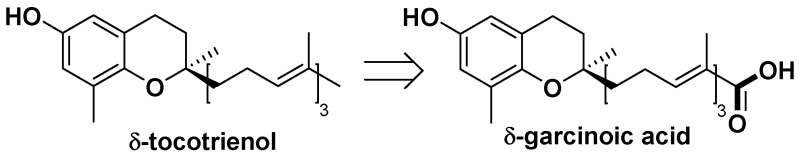


### 4.13. mPGES-1 as a New Target for Garcinoic Acid Isoforms

AlsabilKhaled^1^*Suor-ChererSorphon^1^KoeberleAndreas^2^ViaultGuillaume^1^SchusterDaniela^3^HelesbeuxJean-Jacques^1^StuppnerHermann^3^WerzOliver^2^SeraphinDenis^1^RichommePascal^1^1SONAS, SFR4207 QUASAV, University of Angers, 42 Rue Georges Morel, 49070 Beaucouzé, France2Department of Pharmaceutical/Medicinal Chemistry, Institute of Pharmacy, Friedrich-Schiller-University Jena, Philosophenweg 14, 07743 Jena, Germany3Institute of Pharmacy/Pharmacognosy and Center for Molecular Biosciences Innsbruck, University of Innsbruck, Innrain 80-82, 6020 Innsbruck, Austria*Correspondence: khaledsabil88@yahoo.com

In the course of the Austrian interdisciplinary network project “Drugs from Nature Targeting Inflammation” (DNTI: 2007–2014) (Waltenberger, B., et al. *Monatsh. Chem.* 2016, *147*, 479–491), a comprehensive in silico pharmacophore-based virtual screening of tocotrienol (T3) derivatives isolated from *Garcinia amplexicaulis* (Lavaud, A., et al. *J. Nat. Prod.* 2013, *76*, 2246–2252) was performed. This parallel profiling of T3 derivatives against various anti-inflammatory targets clearly revealed δ and γ-tocotrienolic acids (δ and γ-garcinoic acids, δ and γ-GA) as potential microsomal prostaglandin E synthase-1 (mPGES-1) inhibitors. It then appeared of great interest to evaluate in vitro the capacity of the whole series of GA (i.e., δ, β, γ, and α derivatives) to modulate mPGES-1 activity. This enzyme transforms prostaglandin (PG) H2, the unstable cyclooxygenase-derived oxidation product of arachidonic acid, to PGE2 and might be considered as a key target in inflammation (Koeberle, A., et al. *Biochem. Pharmacol.* 2015, *98*, 1–15). For these investigations, β and α-GA, which have never been isolated from natural sources, were sequentially semisynthesized from δ-GA whereas γ-GA was isolated for the first time from *Garcinia amplexicaulis* stem bark.


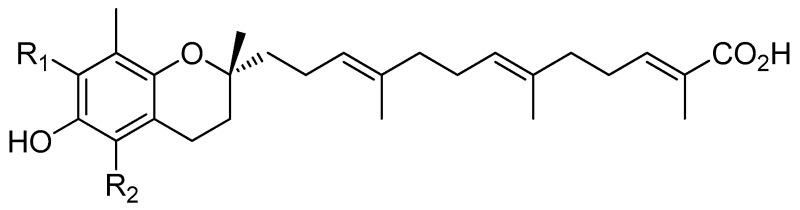


### 4.14. Design, Synthesis and Biological Evaluation of Fluorescent Ligands for MT_1_ and/or MT_2_ Melatonin Receptors

ViaultGuillaume^1^PoupartSéverine^1^MourlevatSophie^2^LagaraineChristine^2^DevavrySéverine^2^LefoulonFrançois^3^BozonVéronique^2^DufournyLaurence^2^DelagrangePhilippe^4^GuillaumetGérald^1^SuzenetFranck.^1^*1ICOA, Université d’Orléans, UMR CNRS 7311, B.P. 6759, 45067 Orléans CEDEX 2, France2PRC, INRA, CNRS, IFCE, Université de Tours, 37380 Nouzilly, France3Technologie SERVIER, 27 rue Vignat, Orléans 45000, France4Institut de Recherche Servier, Sciences Expérimentales, 125 Chemin de Ronde, 78290 Croissy, France*Correspondence: franck.suzenet@univ-orleans.fr

Melatonin (*N*-acetyl-5-methoxytryptamine) is a neurohormone synthesized by the pineal gland during the dark period in all animal species and immediately released. The rhythm of melatonin synthesis is directly controlled by photoperiod. Melatonin secretion both in blood and in cerebrospinal fluid transduces the photoperiodic message to all central or peripheral structures expressing melatoninergic receptors or binding sites, allowing synchronization of several cellular and physiological events to a given day-length. Melatonin rhythm synchronizes a wide range of physiological functions including among others circadian rhythms, seasonal reproduction, immunity etc. Anarchic melatonin secretion also accounts for disorders of sleep/wake rhythms associated or not with depression.


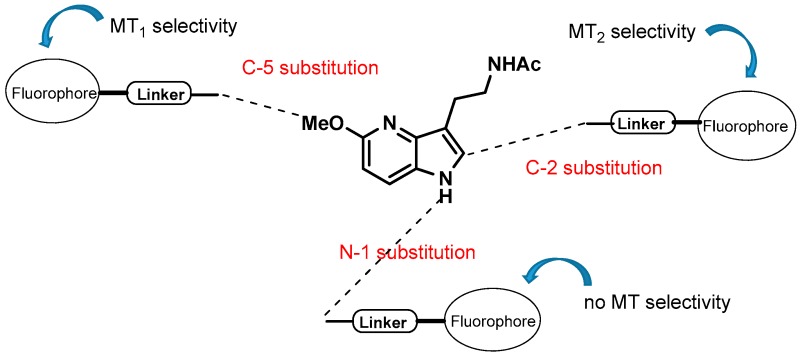


Fluorescent melatoninergic ligands (Viault, G., et al. *RSC Adv.* 2016, *6*, 62508–62521) were designed by associating the 4-azamelatonin ligands with different fluorophores. The ligands show good affinities for MT_1_ and/or MT_2_ receptors and substitution of the fluorophore at positions 2 or 5 of the azamelatonin core had a direct impact on the MT receptors selectivity while grafting the fluorophores on position N-1 produced fluorescent ligands with good affinities for both MT_1_/MT_2_ receptors. The optimal position N-1, C-2 or C-5 on the 4-azamelatonin ligand appeared strongly dependent upon the nature of the fluorophore itself.

## 5. Conclusions

To honor the work of Ph.D. students and young scientists, two awards were attributed for the best oral flash presentation and for the best poster presentation. The first award was attributed to Mary McKee (Department of Chemistry and ABCRF, University College Cork, Western Road, Cork, Ireland) for her presentation entitled “Synthesis and Evaluation of Novel Benzofuran Conjugates as Potential Anticancer Agents”. Alexia Ville (SONAS, SFR4207 QUASAV, University of Angers, France) received the award for the best poster. It was entitled “δ-Garcinoic Acid, a Convenient Natural Precursor to the Attractive δ-Tocotrienol”. 

The 25th Conference of GP2A will take place next summer, the 31^st^ of August and 1^st^ of September 2017 in Liverpool (GB).

